# Midterm prognosis of type B aortic dissection with and without dissecting aneurysm of descending thoracic aorta after endovascular repair

**DOI:** 10.1038/s41598-019-45472-w

**Published:** 2019-06-20

**Authors:** Jian Wang, Jichun Zhao, Yukui Ma, Bin Huang, Ding Yuan, Yi Yang

**Affiliations:** 1 The West China Medical School of Sichuan University, West China Hospital of Sichuan University, Department of Vascular Surgery, Chengdu, 610041 Sichuan China; 2 West China Hospital of Sichuan University, Department of Vascular Surgery, Chengdu, 610041 Sichuan province China

**Keywords:** Aneurysm, Aortic diseases

## Abstract

Few studies support guidelines for the use of thoracic endovascular aortic repair (TEVAR) to address type B aortic dissection (TBAD) coexisting with descending thoracic aortic dissection and aneurysm (dTADA). This cohort study investigated midterm outcomes of TBAD with dTADA (dTADA group, n = 31) and without dTADA (non-dTADA group, n = 98) after TEVAR. Compared with the non-dTADA group, the dTADA group exhibited higher incidences of type Ia endoleak (29.0% vs. 3.1%, *P* < 0.001) and reintervention (16.1% vs. 5.1%, *P* = 0.045). The completely thrombosed rate of the thoracic false lumen was significantly lower in the dTADA group than in the non-dTADA group (45.2% vs. 80.6%, *P* < 0.001). Although the two groups exhibited similar mortality rates, TBAD coexisting with no regressive dTADA after TEVAR was an independent predictor of mortality (HR: 15.52, 95% CI: 1.614–149.233, *P* = 0.018). Moreover, the change percentages of false lumen retraction and true lumen re-expansion in the dTADA group were significantly inferior to those of the non-dTADA group at levels of 4th, 6th, 8th and 10th thoracic vertebra throughout follow-up. In conclusion, in the presence of preexisting dTADA, the failure of the dTADA to regress after TEVAR is associated with lower survival and a higher risk of reintervention.

## Introduction

It has been reported that approximately 14.2–15.7% of aortic dissections are accompanied by descending aortic aneurysms, whereas approximately 1.6–4.9% of descending aortic aneurysms coexist with aortic dissection^1–4^. Therefore, type B aortic dissection (TBAD) with descending aortic aneurysms occurs rarely. Currently, guidelines do not clearly describe a therapeutic strategy for TBAD with descending thoracic aortic dissection and aneurysm (dTADA); instead, they recommend that complicated TBAD should be treated with thoracic aortic endovascular repair (TEVAR) or open repair. Importantly, TBAD coexisting with dTADA is a complicated type of dissection^5^. Guidelines suggest that TEVAR should be performed in cases of aneurysmal dissection of the descending aorta with a maximal diameter ≥5.5 cm and considered for patients with systemic hypertension in a lower threshold of 5 cm, because dissection is associated with significant aortic growth over time^5,6^.

TEVAR is an advanced therapy recommended for the management of complicated type B aortic dissection because it significantly reduces morbidity and mortality compared with open repair^7,8^. Few comparative studies are available regarding TEVAR in the treatment of TBAD with and without dTADA. Hence, the purpose of this study was to evaluate the midterm prognosis of TEVAR in treating TBAD coexisting with dTADA.

## Materials and Methods

### Study population

**Figure 1:**
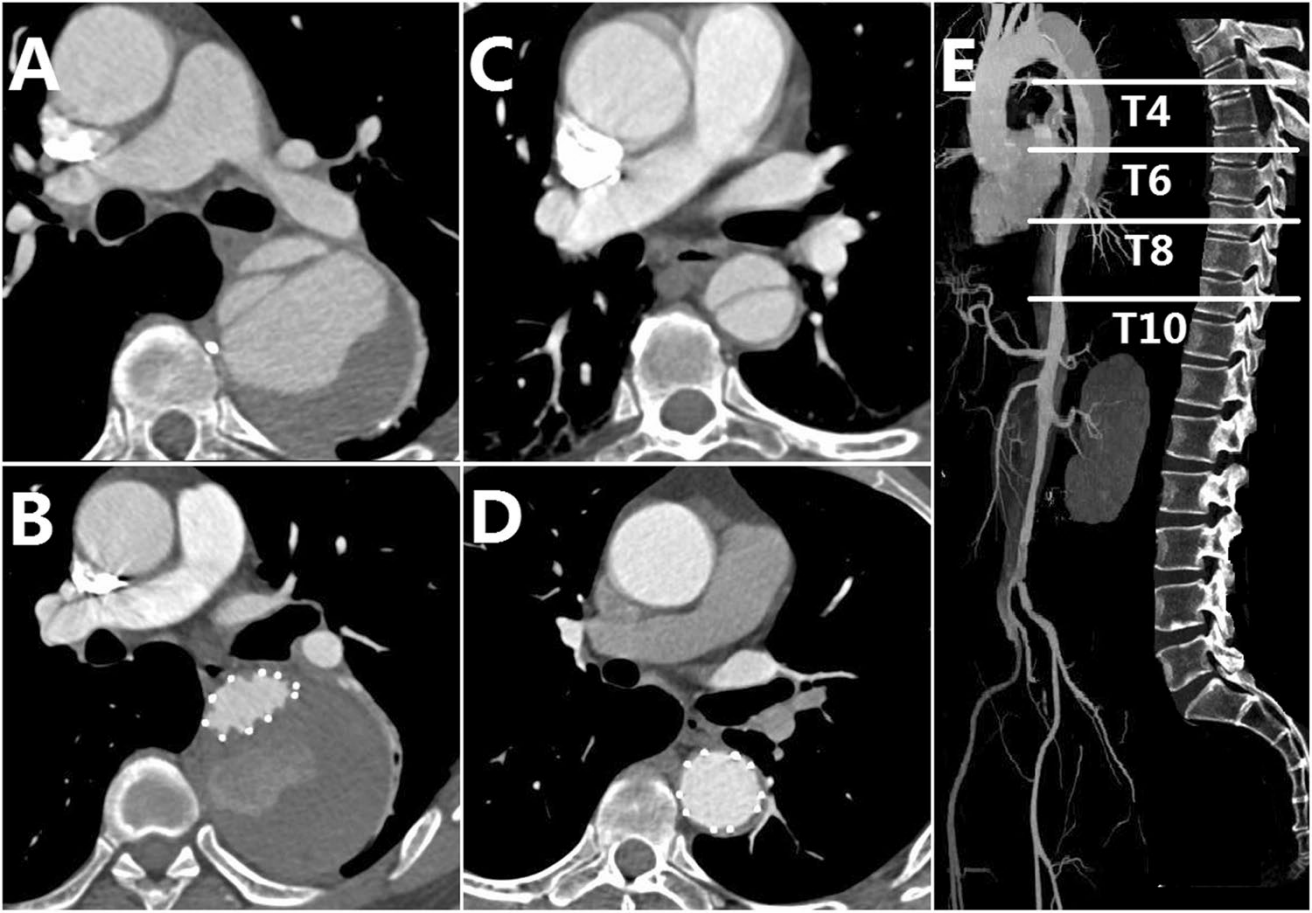
(**A**) Type B aortic dissection with descending thoracic aortic dissection and aneurysm before endovascular repair. (**B**) Patient of (**A**) with partial thrombosis at one year after the operation. (**C**) Another patient with type B aortic dissection alone before endovascular repair. (**D**) Patient of (**C**) with complete regression of false lumen one year after surgery. (**E**) Measurement levels of the fourth thoracic vertebrae (T4), T6, T8, and T10.

From January 2013 to December 2016, a total of 129 consecutive patients with TBAD who underwent TEVAR in our institution were retrospectively reviewed. Patients were divided into two groups (dTADA group, n = 31 and non-dTADA group, n = 98) according to whether preoperative TBAD was accompanied by dTADA (Fig. 1A,B) or was not accompanied by dTADA (Fig. 1C,D). dTADA was defined as a minimum cross-sectional diameter of greater than 5 cm in the proximal descending aortic dissection and was regarded as the criterion for dividing the groups in this study. Definitions of TBAD stages (acute, subacute, and chronic) used in this study were as described in the VIRTUE Registry^9^. The protocol of this study was performed in accordance with the declaration of Helsinki and approved by the Ethics Committee of West China Hospital of Sichuan University. All patients provided written informed consent to undergo all surgical procedures and participate in clinical research.

### TEVAR procedure

TEVAR procedures were performed in a hybrid operating room under general anaesthesia with open femoral access. Intraoperatively, a single dose of intravenous heparin was administered (62.5 U/kg of body weight) before a femoral arteriotomy and half dose of heparin was added after one hour, to achieve systemic anticoagulation (the goal ACT was about twice as large as the basic ACT). According to the preoperative assessment and intraoperative angiography, the thoracic stent graft (Medtronic Valiant and Talent, 28–46 mm in diameter and 150 or 200 mm in length), which was no more than 10% oversized, was guided to the proximal non-dissected aorta and deployed to seal the proximal entry tear. The chimney technique or surgical debranching with proximal device occlusion of the left subclavian arteries (LSA) was routinely performed before the deployment of thoracic stent grafts when LSA was totally covered to acquire an adequate proximal landing zone. If the left common carotid artery (LCCA) was involved by dissection, the double chimney (LCCA and LSA) was performed. Additionally, in the setting of radiological evidence of a serious collapsed true lumen in descending aorta, a restrictive stent (Medtronic Endurant iliac limb, 20–28 mm in diameter and 80 mm in length) as an adjunct to TEVAR was placed distally in the descending aorta prior to TEVAR to prevent distal stent-induced new entry as previously described^10,11^. Intraoperatively, cerebrospinal fluid drainage and embolic protection devices were not routine. In order to prevent spinal ischemia and thromboembolism, these intraoperative details that maintenance of suitable blood pressure (120–140 mmHg), heparinization, reduction of redundant manipulation, accurate deployment of stents were necessary. Additionally, all patients were routinely given with subcutaneous weight-adjusted low-molecular-weight heparin (LMWH) and alprostadil in the postoperative treatment.

### Data collection

All patients received CT angiography (CTA) within 1 month after TEVAR as well as 3 months, 12 months and yearly thereafter. Collected data included perioperative details and postprocedural morbidities of endoleak, stroke, paraplegia, retrograde type A aortic dissection, stent-induced new entry (SINE), postoperative dTADA and reintervention. Preoperative dTADA was defined as TBAD accompanied by dTADA (≥5 cm) before TEVAR. Postoperative dTADA (≥5 cm) and expanding dTADA (>5 cm) were derived from pre-operative dTADA and/or new dilated dTADA. Type Ia endoleak was defined as blood flowing in a false lumen via the proximal fissure between the stent graft and aortic wall. Type Ib endoleak was defined as blood flowing in a thoracic false lumen from the stent-distal entry. Type II endoleak was defined as blood retrograde into a false lumen from the left subclavian artery, intercostal arteries, or aberrant right subclavian artery. Outcome indicators were determined by the medical records and a CTA at the end of follow-up. The mortality assessment was based on the medical records, the CTA follow-up, and a telephone interview. Any patient with sudden death, undetected blood pressure, and radiological evidence prior to death showing a risk factor of dissection rupture was defined as aorta-related mortality.

### Assessment of aortic remodelling

Aortic remodelling was investigated by a true lumen (TL) re-expansion and false lumen (FL) retraction with concomitant thrombosis. In this study, the diameters of FL and TL were measured at the levels of the fourth thoracic vertebra (T4) positioned by the bifurcation of the trachea and at T6, T8, and T10 (Fig. 1E). The percentage of diameter changes were calculated and compared between the two groups. The percentage of diameter change was equal to the diameter of one plane at a follow-up divided by the preoperative diameter of that plane, and minus 100% (Diameter change % = D_Tx post-operation_/D_Tx pre-operation_ − 100%). The morphological characteristics of FL were classified as complete thrombosis, partial thrombosis, and patency.

### Statistical analysis

SPSS 16.0 (SPSS Inc., Chicago, IL) and GraphPad Prism 5.0 (GraphPad Software Inc., La Jolla, CA) were used for analysis and to generate charts. Continuous variables are expressed as the mean ± standard deviation and compared by two-sided t-test or t’-test. Categorical variables were compared by two-sided χ^2^ test or Fisher’s exact test. Covariables associated with postoperative dTADA and mortality were analyzed by Cox regression. *P* < 0.05 was considered statistically significant.

## Results

### Demographics

Perioperative baseline characteristics of patients were similar between the two groups (Table 1). The average diameter of dTADA was 6.17 ± 1.13 cm, and the distance to the left subclavian artery and the celiac trunk were 3.39 ± 2.77 cm and 13.21 ± 6.26 cm, respectively. The patients underwent prior aortic surgeries to extend the proximal landing zone in the treatment of TBAD accompanied by dTADA; these surgeries included the LSA and/or LCA chimney technique (16.2%), bypass surgery (6.5%), LSA occlusion (12.9%), and LSA complete coverage (12.9%). These procedures were not significantly different between the two groups. Additionally, restrictive stent placement was performed at a similar rate in both groups.

**Table 1:** Demographics and medical history.

	dTADA (31)	Non-dTADA (98)	P
Diameter, cm	6.17 ± 1.13	—	—
Distance to the LSA, cm	3.39 ± 2.77	—	—
Distance to the CT, cm	13.21 ± 6.26	—	—
Age, years	51.42 ± 10.4	50.78 ± 10.7	0.770
Sex (female)	3/31(9.7%)	19/98(19.4%)	0.210
Weight, kg	69.10 ± 11.4	72.89 ± 11.9	0.200
Smoking	13/31(41.9%)	44/98(44.9%)	0.772
Drinking	7/31(22.6%)	35/98(35.7%)	0.174
**TBAD stages**			
Acute	16/31(51.6%)	62/98(63.3%)	0.247
Subacute	10/31(32.3%)	29/98(29.6%)	0.778
Chronic	5/31(16. 1%)	7/98(7.1%)	0.159
Hypertension	21/31(67.7%)	73/98(74.5%)	0.461
Diabetes mellitus	0/31(0%)	2/98(2%)	1.000
Cardiac insufficiency	3/31(9.7%)	10/98(10.2%)	1.000
Renal insufficiency	0/31(0%)	4/98(4.1%)	0.572
Stroke	1/31(3.2%)	1/98(1.0%)	0.424
COPD	0/31(0%)	1/98(1.0%)	1.000
Intestinal ischemia	0/31(0%)	2/98(2%)	1.000
Limb ischemia	1/31(3.2%)	8/98(8.2%)	0.686
Restrictive stent	18/31(58.1%)	51/98(52.0%)	0.558
**Chimney**			
LSA	3/31(9.7%)	5/98(5.1%)	0.398
LCA	2/31(6.5%)	5/98(5.1%)	0.674
LCA + LSA	0/31(0%)	2/98(2%)	1.000
LCA to LSA bypass	2/31(6.5%)	6/98(6.1%)	1.000
**Occluder**			
LSA	3/31(12.9%)	5/98(5.1%)	0.217
AA entry	1/31(3.2%)	0/98(0%)	0.385
**LSA coverage**			
Complete	4/31(12.9%)	16/98(16.3%)	0.781
Partial	4/31(12.9%)	20/98(20.4%)	0.349
No	23/31(74.2%)	62/98(63.3%)	0.262

dTADA: descending thoracic aortic dissection and aneurysm. TBAD: Type B aortic dissection. COPD: Chronic obstructive pulmonary disease. LSA: Left subclavian artery. CT: Celiac trunk. LCA: Left carotid artery. AA: Abdominal aorta.

### Follow-up outcomes

Complications are shown in Table 2. The mean follow-up duration was 27.2 ± 16.8 months for the dTADA group and 23.4 ± 11.6 months for the non-dTADA group. Incidences of endoleak were significantly higher in the dTADA group than the non-dTADA group, including type Ia and type Ib endoleaks. A significantly greater number of secondary interventions were observed in the dTADA group than the non-dTADA group. In the dTADA group, a secondary intervention was accomplished for two patients with type Ia endoleak, two patients with type Ib endoleak and one patient with a coexisting case of type Ia endoleak and type Ib endoleak. In the non-dTADA group, reintervention was performed for two patients with SINE, two patients with iliac artery aneurysm and one patient with type Ia endoleak. Moreover, the morbidities of postoperative dTADA and expanding dTADA were also higher in the dTADA group than the non-dTADA group. The regressive rate of preexisting dTADA is 35.5% (31-20/31) after endovascular repair. Aorta-related mortality and overall mortality were not significantly different between the two groups. No 30-day death was observed in either group.

**Table 2:** Morbidities and mortality.

	dTADA (31)	Non-dTADA (98)	P
Follow up, months	27.2 ± 16.8	23.4 ± 11.6	0.232
TAFL complete thrombosis	14/31(45.2%)	79/98(80.6%)	**0.000**
TAFL partial thrombosis	17/31(54.8%)	19/98(19.4%)	**0.000**
TAFL patency	0/31(0%)	0/98(0%)	1.000
AAFL complete thrombosis	6/31(19.4%)	18/98(18.4%)	0.902
AAFL partial thrombosis	16/31(51.6%)	53/98(54.0%)	0.810
AAFL patency	9/31(29.0%)	27/98(27.6%)	0.873
AADA	6/31(19.4%)	8/98(8.2%)	0.081
Type Ia endoleak	9/31(29.0%)	3/98(3.1%)	**0.000**
Type Ib endoleak	12/31(38.2%)	17/98(17.3%)	**0.024**
Type II endoleak	3/31(9.7%)	2/98(2.0%)	0.089
RAAD	1/31(3.2%)	1/98(1.0%)	0.424
SINE	4/31(12.9%)	5/98(5.1%)	0.217
30 day mortality	0/31(%)	0/98(%)	1.000
Aorta related mortality	2/31(6.5%)	2/98(2.0%)	0.244
Total mortality	2/31(6.5%)	4/98(4.1%)	0.630
Reintervention	5/31(16.1%)	5/98(5.1%)	**0.045**
Paraplegia	0/31(0%)	0/98(0%)	1.000
Stroke	0/31(0%)	0/98(0%)	1.000
Postoperative dTADA	19/31(61.3%)	2/98(2.0%)	**0.000**

dTADA: descending thoracic aortic dissection and aneurysm. TAFL: Thoracic aortic false lumen. AAFL: Abdominal aortic false lumen. AADA: Abdominal aortic dissecting aneurysm. RAAD: Retrograde type A aortic dissection. SINE: Stent-induced new entry.

### Predictor of mortality

Significant factors in Table 2 were selected as covariates to predict mortality. Univariate predictors of mortality are shown in Table 3. Results revealed that the postoperative dTADA was an independent predictor of mortality. Furthermore, multivariate regression found that postoperative dTADA is most often a result of preoperative dTADA (Table 4).

**Table 3:** Univariate Cox regression analyzed predictor of mortality.

Univariate		
	HR (95% CI)	*P*
Preoperative dTADA	2.986 (0.420–21.206)	0.274
Postoperative dTADA	15.52 (1.614–149.233)	**0.018**
Type Ia endoleak	3.352 (0.349–34.235)	0.295
Type Ib endoleak	3.438 (0.484–24.410)	0.217
Reintervention	0.044 (0.000–2.404)	0.692
TAFL complete thrombosis	0.128 (0.013–1.233)	0.075
TAFL partial thrombosis	7.794 (0.811–74.935)	0.075

HR: Hazard ratio. CI: Confidence interval. dTADA: descending thoracic aortic dissection and aneurysm. TAFL: Thoracic aortic false lumen.

**Table 4:** Cox regression analyzed predictors of the postoperative dTADA.

	Univariate		Multivariate	
	HR (95% CI)	*p*	HR (95% CI)	*p*
Preoperative TADA	24.954 (5.796–107.429)	**0.000**	18.250 (4.076–81.708)	**0.000**
Type Ia endoleak	6.875 (2.816–16.781)	**0.000**	1.772 (0.506–6.205)	0.371
Type Ib endoleak	2.652 (1.054–6.675)	**0.038**	1.654 (5.411–31.289)	0.494
Type II endoleak	2.899 (1.469–9.929)	0.160		
SINE	2.140 (0.618–7.415)	0.230		
Reintervention	1.111 (0.257–4.800)	0.888		
TAFL thrombosis	0.207 (0.086–0.496)	**0.000**	0.457 (0.100–2.086)	0.312
TAFL partial thrombosis	4.833 (2.016–11.582)	**0.000**	2.191 (1.457–5.779)	0.312

HR: Hazard ratio. CI: Confidence interval. dTADA: descending thoracic aortic dissection and aneurysm. SINE: Stent-induced new entry. TAFL: Thoracic aortic false lumen.

### Aortic remodelling

Morphological remodelling of the thoracoabdominal dissection is summarized in Table 2. A lower frequency of complete thrombosis and a higher proportion of partial thrombosis for thoracic FL were observed in the dTADA group compared with the non-dTADA group. A similar proportion of abdominal FL thrombosis was noted between the two groups.

**Figure 2:**
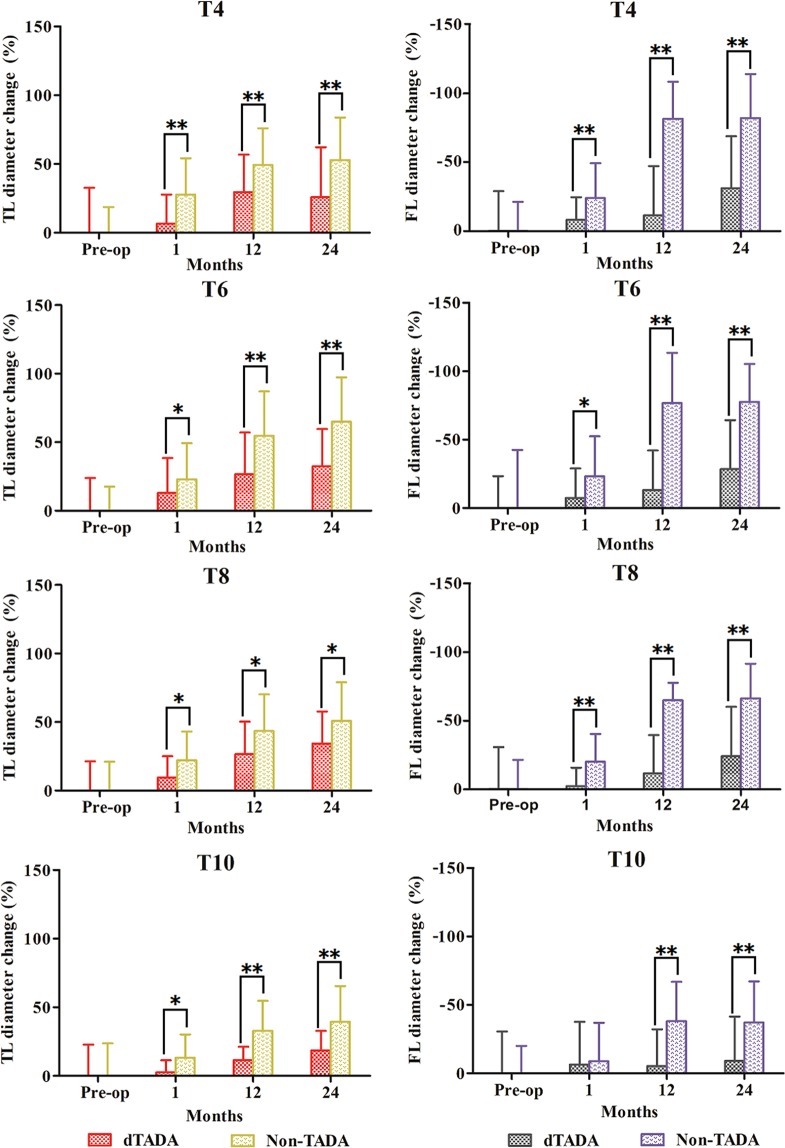
The percentage of diameter change was equal to the diameter of one plane at a follow-up divided by the preoperative diameter of that plane, and minus 100% (Diameter change % = D_Tx post-operation_/D_Tx pre-operation_ − 100%). Diameter change % of the true lumen (TL) and false lumen (FL) were compared at levels of the fourth thoracic vertebrae (T4), T6, T8 and T10 between the dTADA groups and non-dTADA groups. Pre-op is an abbreviation of pre-operation. Minus (−) represents a trend of FL shrinkage. Asterisk (*) represents *P* < 0.05, the double asterisk (**) represents *P* < 0.01.

Diameter changes of the TL and FL at levels of T4, T6, T8 and T10 are plotted in Fig. 2. TL re-expansion and FL shrinkage were observed in each group during the follow-ups. Results showed that TL diameter changes were significantly greater in the non-dTADA group compared with the dTADA group at the end of follow-up: at the T4 level, 52.9 ± 30.9% vs. 25.8 ± 36.4%, P = 0.009; T6 level, 64.9 ± 32.4% vs. 32.4 ± 27.1%, P = 0.002; T8 level, 50.8 ± 28.2% vs. 34.3 ± 23.4%, P = 0.047; and T10 level, 39.3 ± 26.1% vs. 18.5 ± 14.3%, P > 0.001). Similarly, the change of FL shrinkage in the non-dTADA group was significantly more than that in the dTADA group at the end of follow-up: at the T4 level, −81.9 ± 17.1% vs. −31.1 ± 37.6%, P > 0.001; T6 level, −74.6 ± 22.7% vs. −28.4 ± 31.8%, P = 0.001; T8 level, −66.1 ± 22.5% vs. −24.0 ± 31.0%, P = 0.003; and T10 level, −37.1 ± 30.1% vs. −9.1 ± 32.3%, P = 0.008).

## Discussion

Descending aortic aneurysm (guideline recommend: diameter ≥55 mm^5^) can be earlier addressed by the TEVAR procedure. However, patients with descending aortic aneurysm are often asymptomatic and occasionally present with acute aortic dissection. Accumulating evidence has shown that 14.2–15.7% of aortic dissections are accompanied by descending aortic aneurysm and 1.6–4.9% of descending aortic aneurysm are accompanied by aortic dissection^1–4^. However, an incidence up to 21% of coexistent aortic aneurysms were reported in 195 patients who underwent type B dissection^12^. We found coexistent true descending aortic aneurysm in 31/129 (24%) of patients with TBAD. It was reported that there is a rapid increase in the risk of dissection when the aortic diameter is 60 mm for the ascending aorta and 70 mm for the descending aorta^5,13^. Moreover, dissection can still occur in patients with a small aorta. Currently, the absence of studies supports the guideline’s suggestion of using TEVAR in the treatment of TBAD coexisting with dTADA. In our study, we presented midterm outcomes to better understand the potential role of TEVAR in this condition. We found that TBAD coexisting with dTADA was associated with higher incidences of partial FL thrombosis, endoleak, secondary intervention, postoperative dTADA and expanding dTADA (Table 2). Previous studies mentioned that a relatively high incidence of type I endoleak was observed after emergent endovascular repair of descending aortic aneurysm^14^ and that the degree of fusiform dilatation (≥40 mm) of the proximal descending aorta had significantly more aortic events, including expanding dTADA and partial FL thrombosis in patients with type B aortic dissection^15,16^. The mean diameter of dTADA was 6.17 ± 1.13 cm in this study, which obviously exceeded the diameter of 40 mm. The progression of FL thrombosis was relatively slower in the dissecting aneurysmal sac, which may result in a higher intracapsular pressure in the aneurysmal FL than in the non-aneurysmal FL. The higher intracapsular pressure hindered the stent graft expanding to its definitive dimension and further induced aneurysm degeneration. According to Fig. 2, the change percentage of TL and FL regression was significantly lower in the dTADA group than the non-dTADA group at levels of T4, T6, T8 and T10 throughout the follow-up. As a result, type Ia and/or type Ib endoleak would occur. A biologic model of TBAD proved that the FL pressure is reduced with proximal tear occlusion and increased with distal tear occlusion^17^. The single type Ia endoleak may prominently increase FL pressure. Additionally, type Ia or II endoleak, which is regarded as the inflow route, and type Ib endoleak or intimal entry, which is regarded as the outflow route, may construct a communication channel between the FL and TL^18^, which results in partial FL thrombosis, no FL shrinkage, and even FL expansion or rupture. It seems that endoleak and the presenting pathology play complementary roles in promoting the aneurysmal degeneration of false lumen. From Table 2, we calculate that the incidence of postoperative dTADA is 64.5% (20/31), and its expansion event is 16.1% (5/31) in patients with dTADA after endovascular repair. If a FL continues to expand with time, reintervention procedures are inevitable for patients.

Table 2 shows a similar incidence of aorta-related death between the two groups. The results showed that the reintervention rate was significantly higher in the dTADA group. In addition, some FL regression in the TBAD coexisting with dTADA after endovascular repair may decrease patient mortality. It can be inferred from Table 2 that the regressive rate of dTADA is 35.5% (31-20/31) after TEVAR. In this study, two aortic deaths were found in the patients who had experienced LSA chimney, but one patient with type Ia endoleak was related to the failure regression of preoperative dTADA after endovascular repair, another patient with type Ib endoleak was related to an expanding dTADA that was newly formed after TEVAR. A restrictive stent as an adjunct to TEVAR was deployed in the 69 cases, and two aortic deaths were observed in this subgroup. Hence, restrictive stent had no influence on aortic mortality (2/69 vs. 2/60). Though the mortality rate is similar between the dTADA group and non-dTADA group, Cox regression indicates that postoperative presence of dTADA was an independent predictor of mortality in Table 3. The above explanation mentioned that the incompletely thrombosed FL with a higher pressure may cause FL expansion and even rupture. dTADA may accelerate FL dilation and rupture. Moreover, 95.2% (20/21) of postoperative dTADA originated from preoperative dTADA. Table 4 also disclosed that preoperative dTADA is an independent predictor of the postoperative dTADA. Therefore, preoperative TBAD accompanied by dTADA remained a key threat to the long-term survival of patients after TEVAR.

Previous studies have indicated that the key factors leading to aortic remodelling are TL re-expansion and FL retraction concomitant with thrombosis^19,20^. Our results showed that the dTADA group presented a significantly lower proportion of complete FL thrombosis than the non-dTADA group in the thoracic aortic segment (Table 2). Furthermore, the change percentage of FL retraction and TL re-expansion in the dTADA group were significantly inferior to the non-dTADA group at the 4th, 6th, 8th and 10th levels throughout the follow-ups (Fig. 2). The communicative channel between the FL and TL may lead to partial FL thrombosis, retarded FL shrinkage, and potential FL enlargement. Simultaneously, TL re-expansion may be slowed down owing to a higher FL pressure. Moreover, a larger FL volume in the dTADA group undoubtedly retarded the progress of FL shrinkage and thrombosis. Therefore, the effect of TEVAR on favorable aortic remodelling in the treatment of TBAD coexistent with dTADA was inferior. In the study, 18 restrictive stents were deployed in the dTADA group, among them, 8 cases thoracic FL were complete thrombosis. Other patients who received standard TEVAR in the dTADA group included 6 thoracic FL with complete thrombosis. Hence, restrictive stent had no influence on aortic remodelling in the TBAD with dTADA cohort (8/18 vs. 6/13). This may be related to the length (80 mm) of the restrictive stent. Moreover, more than 50% length of the restrictive stent overlapped with the proximal stentgraft.

The limitations of this study included its uncontrolled, retrospective design and relatively small sample size. The mechanism explains why TBAD with dTADA caused significant stent-related morbidities, and partial FL thrombosis needs to be further elucidated by haemodynamic methods.

## Conclusions

In the presence of pre-existing dTADA, the failure of the aneurysm to regress after TEVAR is associated with lower survival and a higher risk of reintervention. Patients with this coexisting condition after TEVAR might benefit from closer radiologic surveillance.

## Data Availability

All authors declare that materials, data and associated protocols were available to readers without undue qualifications in this study.
